# Molecular Basis of Coupled Transport and Anion Conduction in Excitatory Amino Acid Transporters

**DOI:** 10.1007/s11064-021-03252-x

**Published:** 2021-02-15

**Authors:** Claudia Alleva, Jan-Philipp Machtens, Daniel Kortzak, Ingo Weyand, Christoph Fahlke

**Affiliations:** 1 Institute of Biological Information Processing, Molekular- und Zellphysiologie (IBI-1), Forschungszentrum Jülich, Jülich, Germany; 2 Institute of Clinical Pharmacology, RWTH Aachen University, Aachen, Germany

**Keywords:** Glutamate transporter, Coupled transport, Transport stoichiometry, Induced fit, Conformational selection, Dual function protein, EAAT anion channel, Glt_Ph_, Hairpin 2, MD simulations, Fluorescence spectroscopy, Tryptophan

## Abstract

Glutamate is the major excitatory neurotransmitter in the mammalian central nervous system. After its release from presynaptic nerve terminals, glutamate is quickly removed from the synaptic cleft by excitatory amino acid transporters (EAATs) 1–5, a subfamily of glutamate transporters. The five proteins utilize a complex transport stoichiometry that couples glutamate transport to the symport of three Na^+^ ions and one H^+^ in exchange with one K^+^ to accumulate glutamate against up to 10^6^-fold concentration gradients. They are also anion-selective channels that open and close during transitions along the glutamate transport cycle. EAATs belong to a larger family of secondary-active transporters, the SLC1 family, which also includes purely Na^+^- or H^+^-coupled prokaryotic transporters and Na^+^-dependent neutral amino acid exchangers. In recent years, molecular cloning, heterologous expression, cellular electrophysiology, fluorescence spectroscopy, structural approaches, and molecular simulations have uncovered the molecular mechanisms of coupled transport, substrate selectivity, and anion conduction in EAAT glutamate transporters. Here we review recent findings on EAAT transport mechanisms, with special emphasis on the highly conserved hairpin 2 gate, which has emerged as the central processing unit in many of these functions.

## Transport Functions of EAAT Glutamate Transporters

The excitatory amino acid transporters (EAATs) are the predominant glutamate transporters in the mammalian brain [1]. They were first studied in detail by Baruch Kanner and colleagues in experiments using radiotracer flux experiments and membrane vesicles from the mammalian brain [2–5]. In his seminal work, Baruch described the key features of EAAT function: stereoselective glutamate uptake and the coupling of glutamate transport to the inward movement of at least two Na^+^, with obligatory K^+^ dependence. He also established the first kinetic transport model [5], containing distinct Na^+^ and K^+^ hemicycles; it is still in use, with only minor modifications.

Baruch headed one of the three groups that almost simultaneously identified the amino acid sequences of major glutamate transporters. In 1992, Stoffel and colleagues [6] reported the amino acid sequence of a glycoprotein (GLAST) that was co-purified during the isolation of the UDP-galactose:ceramide galactosyltransferase from rat brain. The protein sequence was similar to those of bacterial transport proteins, and heterologous expression in *Xenopus* oocytes resulted in radioactive glutamate uptake by injected oocytes. Two weeks later, Baruch´s group reported the isolation of a complementary DNA clone encoding the glial glutamate transporter GLT-1 [7], and Kanai and Hediger reported expression cloning of EAAC1 from rabbit small intestine [8]. Subsequently, Amara and colleagues [9] cloned the three human homologs and introduced the EAAT nomenclature by naming them EAAT1, EAAT2, and EAAT3. Homology cloning of EAAT4 cDNA from the cerebellum [10] and of EAAT5 cDNA from the retina [11] completed the mammalian EAAT family. Identification of the protein sequences was the first step toward understanding EAAT function at the molecular level and permitted heterologous expression and high-resolution electrical recording of EAAT currents, genetic modification in animal models, and the linkage of glutamate transporter dysfunction to inherited diseases.

Voltage clamp analysis of injected *Xenopus* oocytes or transfected mammalian cells provided insights into EAAT function at unprecedented accuracy [12–15]. Comparison of glutamate-elicited currents in oocytes expressing EAAT1, EAAT2, or EAAT3 [16] led to the discovery of an EAAT-mediated anion current that is not thermodynamically coupled to amino acid transport. EAAT anion currents largely exceeded transport currents in EAAT4 and EAAT5 [10, 11, 17, 18]. Noise analysis of glutamate-activated current in isolated photoreceptors from the tiger salamander estimated unitary currents that were well above the limits of transporter-mediated fluxes [19], thus providing compelling evidence that EAATs are not only secondary-active glutamate transporters, but can also function as anion channels [20].

In electrogenic transporters, the transport stoichiometry predicts the current reversal potential as a function of the intra- and extracellular ion concentrations. Measurements of transport reversal potentials under various ionic conditions unambiguously demonstrated that EAAT1, EAAT2, and EAAT3 transport three Na^+^ and one H^+^ along with glutamate, in exchange for one K^+^ [13, 21, 22], indicating that EAATs have the most complex transport stoichiometry of all secondary active transporters. A comparison with prokaryotic homologs that couple three Na^+^ to transport of the amino acid substrate [23] illustrates that K^+^ coupling was a late evolutionary addition; however, the physiological importance of this optimization mechanism remains unclear. Astrocytes are most important for glutamate uptake in the mammalian brain. The resting potential of astrocytes is the K^+^ equilibrium potential, at which the driving forces for K^+^-coupled and K^+^-independent transporters are identical. Notably, K^+^ coupling increases the driving force in cells that have membrane potentials positive to the K^+^ equilibrium potential.

## Cellular Roles of Excitatory Amino Acid Transporters

Mammalian EAATs transport glutamate into glial, neuronal, and epithelial cells. EAAT1 and EAAT2 are mainly expressed in glial cells [24]; however, recent data demonstrated that EAAT2 is also present in presynaptic nerve terminals [25, 26]. Whereas the genetic ablation of EAAT2/GLT-1 in mice resulted in lethal spontaneous seizures [27], EAAT1/GLAST knockout led only to motor discoordination and increased susceptibility to cerebellar injury, illustrating a less important role of this isoform in glutamate homeostasis [28]. EAAT3/EAAC1 is not only expressed in neuronal cells, but also in epithelial cells, in which it serves as glutamate uptake system in the kidney and the gastrointestinal tract. Mice lacking EAAT3/EAAC1 exhibit dicarboxylic aminoaciduria owing to impaired amino acid uptake in the proximal tubule [29]. EAAT4 is expressed predominantly in the cerebellum, where it seems to regulate Purkinje cell activity and motor behavior [30]. EAAT5 has been proposed to function as a presynaptic glutamate-gated chloride channel in the retina, where it mediates a negative feedback mechanism for glutamate release [31, 32]. However, no EAAT5 knockout animal has yet been reported. Taken together, these experiments demonstrate that EAAT2 is indispensable for extracellular glutamate homeostasis in the mammalian brain, whereas the other isoforms have only modulatory functions.

The linkage of human genetic diseases to genes encoding EAAT1 (*SLC1A3*), EAAT2 (*SLC1A2*) and EAAT3 (*SLC1A1*) revealed additional roles of EAATs in cell and organ physiology. Mutations in *SLC1A3* cause episodic ataxia type 6 (EA6), a genetic condition characterized by ataxia, epilepsy and hemiplegia*.* The first reported patient with a disease-associated *SLC1A3* mutation was found to be heterozygous for a proline to arginine substitution at position 290 (P290R) of EAAT1 [33]. This amino acid substitution impairs the glutamate transport rate, but increases anion channel activity in heterologous expression systems [34, 35]. In a transgenic mouse model (*Slc1a3*^*P290R/*+^) that has neurological symptoms closely resembling the human disease, ataxia is caused by Bergmann glia apoptosis during infancy and consequent cerebellar degeneration [36]. Bergmann glia apoptosis is triggered by cell shrinking due to increased glutamate-activated Cl^−^ efflux and is thus a direct consequence of gain of function in the P290R EAAT1 anion channel. EAAT1 anion channels appear to be a main determinant in setting the resting [Cl^−^] in Bergmann glia cells of wildtype (WT) mice [37]. Although enhanced anion channel function is the main factor in EA6 pathogenesis in this particular case, a later analysis did not reveal similar functional changes for seven other *SLC1A3* mutations in EA6 patients [38]. In addition to episodic ataxia, migraine [39], Tourette syndrome [40], and attention deficit hyperactivity disorder and autism [41] have been associated with *SLC1A3* mutations.

*SLC1A*2 mutations that predict amino acid exchanges G82R, L85P, or P289R in EAAT2 were reported in three extraordinarily severe cases of epilepsy [42, 43]. The functional consequences of these mutations have not been studied; thus, it is unclear how these mutations affect EAAT2 functions and cause hyperexcitability in the affected individuals. Lastly, two cases of human dicarboxylic aminoaciduria were caused by mutations in *SLC1A1*: a three base pair deletion (c.1184–1186delTCA) and a base pair exchange causing the R445W mutation in EAAT3 [44]. These disease-causing *SLC1A1* mutations illustrate the importance of EAAT3 for amino acid reabsorption in the kidney.

## Structural Basis of Glutamate Transport

**Fig. 1:**
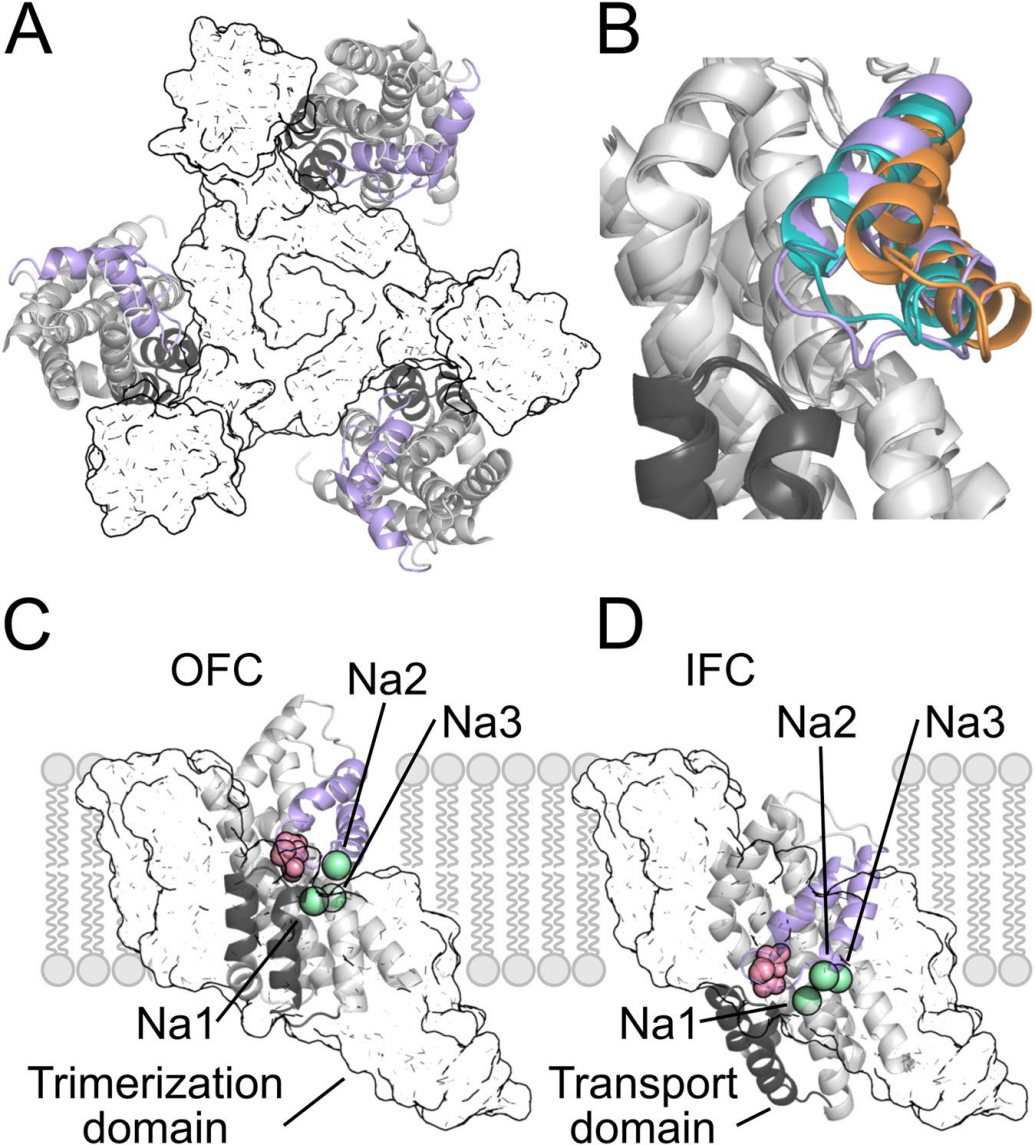
Structures of outward- and inward-facing conformations of Glt_Ph_. **a** Structure of a Glt_Ph_ trimer (PDB ID: 4OYE) in top view (surface, trimerization domains; cartoon, transport domains with HP1 and HP2 highlighted in gray and black/violet, respectively). **b** Close-up of the binding pocket with HP2 in the closed (violet, PDB ID: 4OYE), intermediate (teal, PDB ID: 4OYF), and open (orange, PDB ID: 7AHK) conformations. **c, d** Glt_Ph_ outward-facing (OFC; PDB ID: 2NWX) (**c**) and inward-facing (IFC; PDB ID: 3KBC). **d** conformations in the fully-bound (Na1–3) state in sideview (pink spheres, bound aspartate; green spheres, Na^+^ ions). The Na3-bound ion was not resolved in these structures, and its position was inferred from the Na^+^-only bound Glt_Ph_ structure (PDB ID: 7AHK)

The first three-dimensional structure of a SLC1 transporter was determined for Glt_Ph_, an EAAT homolog of the prokaryote *Pyrococcus horikoshii* [45]. Glt_Ph_ is assembled as a trimer, with each protomer consisting of eight transmembrane helices (TMs) and two helical hairpin loops (HP1 and HP2). Each protomer contains two distinct domains: the trimerization domain, which mediates inter-subunit interactions, and the transport domain, which harbors the ion- and substrate binding sites (Fig. 1a). Crystal and cryo-electron microscopy structures of Glt_Ph_ and of another prokaryotic homolog Glt_Tk_ [45–52] captured the transporter in multiple conformations. For Glt_Ph_, these include the substrate-bound [45], the TBOA-bound [46] and the Na^+^-only bound [49] outward-facing conformation, an intermediate conformation [51], and the substrate-bound inward-facing conformation [50]. Crystal Glt_Tk_ structures were the first to reveal the apo [47] and the fully bound [52] outward-facing conformations, which defined the substrate-binding pocket and all Na^+^-binding sites (including the elusive Na3 binding site). Furthermore, cryo-EM provided Glt_Tk_ structures in various conformational states, including a Na^+^-only bound inward-facing [48] state. The substrate binds between the tips of HP1 and HP2: the different structures exhibit marked variability in the position of HP2, illustrating ion-induced conformational rearrangements of this structural element (Fig. 1b).

A comparison of Glt_Ph_ structures in outward-facing (OFC) [45, 46], inward-facing (IFC) [50], and intermediate (IC) [51] conformations revealed that large-scale (~ 18 Å) rotational/translational movements of the transport domain along the trimerization domain are the basis of substrate and Na^+^ transmembrane ion transport (Fig. 1c, d). Earlier bioinformatic analyses predicted correctly that such elevator rigid-body transmembrane motion of a mobile domain relative to a static domain is the basis of alternating accessibility [53]. Recently, the crystal structure of thermostabilized human EAAT1 [54] and the cryo-electron microscopy structures of the alanine/serine/cysteine/threonine transporter ASCT2 in several conformations [55–57] illustrated structural conservation of the Glt_X_ fold during evolution and confirmed the validity of the elevator transport mechanism.

## HP2 Flexibility is a Key Determinant of Na^+^-Substrate Coupling in Glutamate Transporters

HP2 controls accessibility of the ligand-binding pocket as an extracellular and intracellular gate [46, 57]. Since its open conformation is sterically incompatible with translocation of the transport domain [46, 47, 52, 58], the opening–closing transitions of HP2 also control the translocation process [48, 58]. The competitive inhibitor TBOA blocks transport by locking the transporter in an OFC with an open HP2 [46, 54] and reduces the occupation time of the intermediate state [59].

Glutamate uptake is coupled to the symport of three Na^+^, making the [Na^+^] gradient the most important driving force for synaptic glutamate clearance. The three Na^+^ bind to three different Na^+^-binding sites: Na1 is located below the substrate-binding pocket and thus needs to be occupied prior to substrate binding, whereas Na2 is formed by the closed HP2 and TM7 and can only be occupied upon the closure of HP2 after substrate binding [46]. The third Na^+^-binding site, Na3, was initially proposed in computational studies [60] and recently identified in a crystal structure of fully-bound Glt_Tk_ [52] and in a Glt_Ph_ structure in the Na^+^-only bound state [49].

**Fig. 2:**
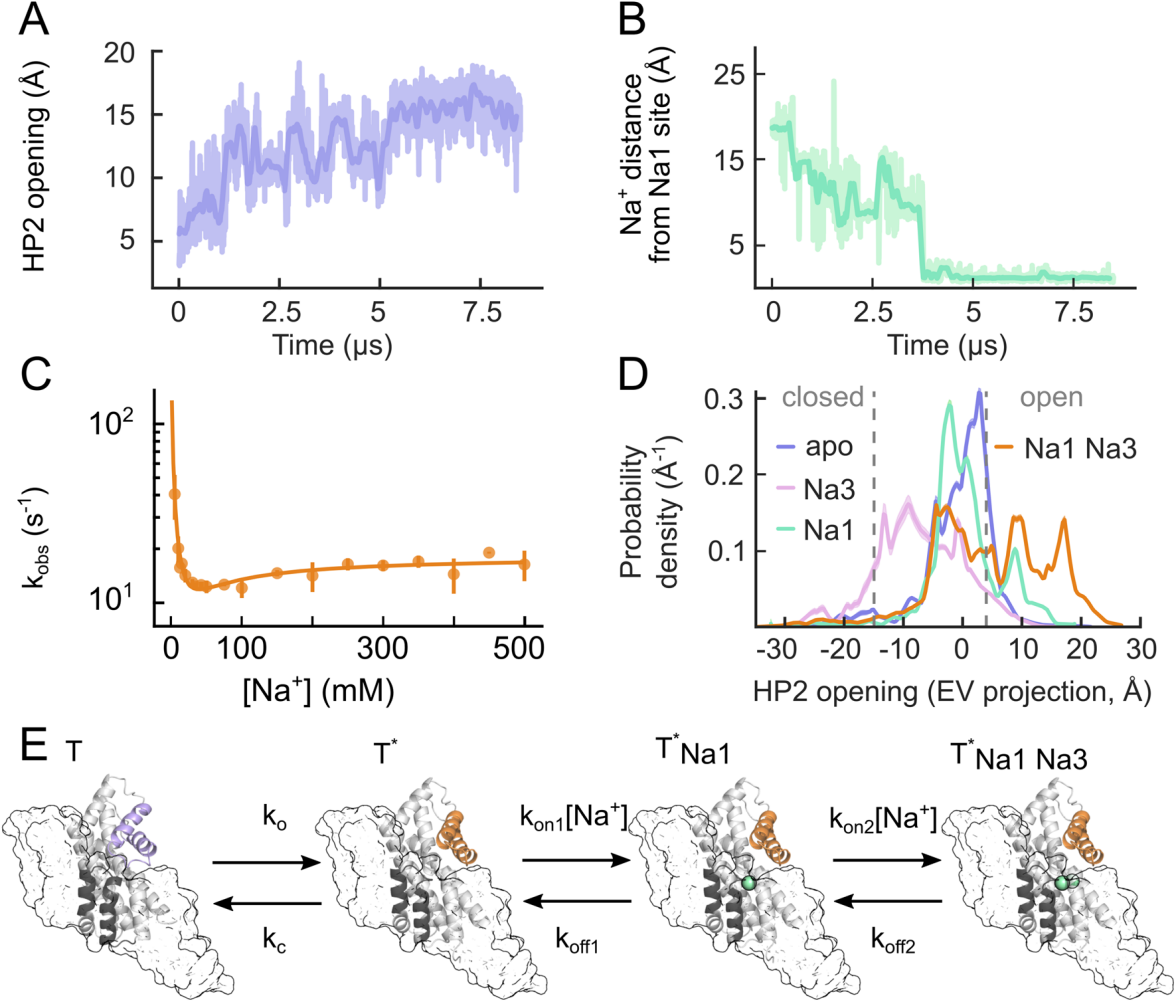
Na^+^-dependent gate dynamics in Glt_Ph_. **a**, **b** Time courses of HP2 opening (measured as the distance between S279 and G354, located on the tips of HP1 and HP2, respectively) (**a**) and of the distance of the closest Na^+^ ion from the Na1 site (**b**) in a representative all-atom MD simulation of Glt_Ph_ in an outward-facing, gate-closed *apo* state. **c** The k_obs_ for rapid Na^+^ application to detergent-solubilized F273W Glt_Ph_ in stopped-flow fluorescence experiments at 20 °C and corresponding fits of the kinetic model described in (**e**). **d** Probability densities for HP2 opening as a function of Na1- and Na3-site occupancies, as obtained by umbrella sampling simulations (EV, eigenvector). **e** Four-state model describing Na^+^ binding to the *apo* transporter. T and T* indicate the closed and open states of HP2, respectively. Figure partially reprinted from [49]

**Fig. 3:**
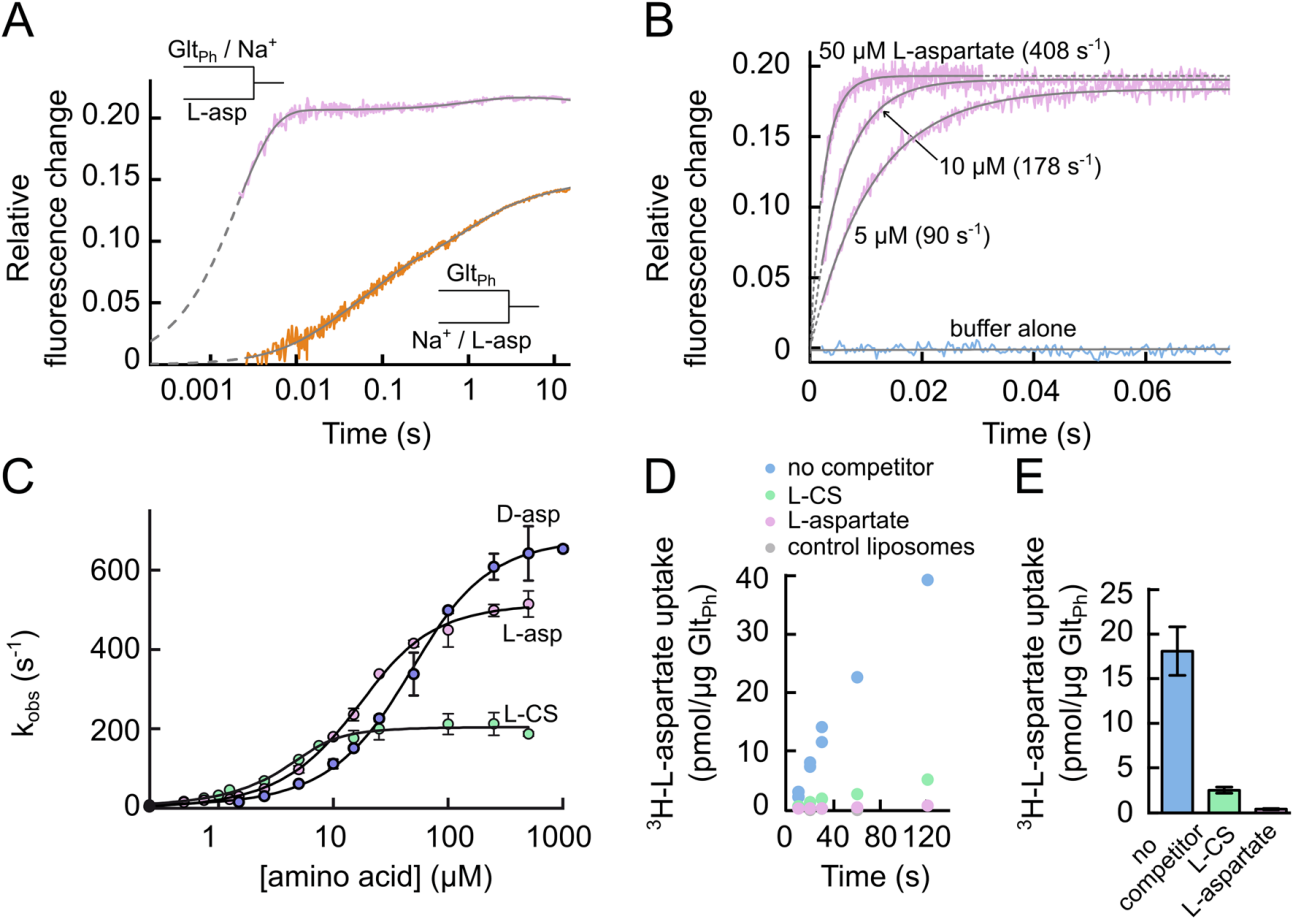
Induced fit substrate association with Glt_Ph_. **a** Tryptophan fluorescence transients upon rapid mixing of 500 µM l-aspartate to L130W Glt_Ph_ pre-equilibrated with 250 mM NaCl (upper curve) or upon simultaneous mixing of 500 µM l-aspartate and 250 mM NaCl with empty L130W Glt_Ph_ (lower curve). NaCl was substituted with equimolar ChoCl. Colored lines are experimental data, and fits to sums of exponential functions are in gray. **b** Tryptophan fluorescence transients after rapid mixing of L130W Glt_Ph_ in 200 mM NaCl with various [l-aspartate]. Experimental data are in colors, and fits to the sum of two exponential functions or to a linear function (trace of buffer alone) are in gray. For each trace, the fitted value of k_obs_ is given. **c** Amino acid concentration dependence of k_obs_, obtained as shown in (**b**). **d** Left: Time course of ^3^H-l-aspartate (100 nM) uptake, as driven by an inwardly directed [Na^+^] gradient in the absence or presence of l-cysteine sulfinic acid (l-CS) and l-aspartate (10 µM each). The first 120 s of a representative experiment are shown, with Glt_Ph_-free liposomes as control. **e** Initial uptake rates in the absence or presence of competitor (mean ± SEM, three experiments). Figure partially reprinted from [64]

In unguided atomistic molecular dynamics (MD) simulations of the outward-facing *apo* Glt_Ph_, HP2 was found to be intrinsically flexible, resulting in spontaneous transitions between the open and closed conformations (Fig. 2a) [49]. In these simulations, Na^+^ binds spontaneously after HP2 opening and subsequent hydration of the Na1 site (Fig. 2b). Occupation of the Na3 site was not observed in unguided MD simulations on timescales up to 10 µs, suggesting that Na3 association is rate-limiting for Na^+^-induced conformation changes (see below, Fig. 3).

WT Glt_Ph_ completely lacks tryptophan residues, and tryptophan insertion at position 273 enabled conformational changes upon Na^+^ binding to be followed using tryptophan fluorescence spectroscopy [61]. W273 reports on the association of Na^+^ and aspartate with opposing changes in fluorescence intensity. Association with Na^+^ and aspartate have opposite effects on HP2 [62]. This finding, together with the differential water accessibility of this residue observed in MD simulations [49], demonstrates that W273 directly reports on HP2 opening and closing. Fast stopped-flow application of Na^+^ to solubilized F273W Glt_Ph_ triggers an increase in fluorescence, with observed rate constant (k_obs_) decreasing as the [Na^+^] rises to ~ 50 mM and then increasing to a plateau at higher Na^+^ concentrations (Fig. 2c). The initial decrease in the k_obs_ demonstrates that a Na^+^-independent conformational change occurs before Na^+^ binding, which resembles a conformational selection mechanism [63]. Subsequent acceleration of k_obs_ with increasing [Na^+^] reveals an additional Na^+^-dependent process. Therefore, Na^+^ binding to *apo* Glt_Ph_ can be described by a state diagram in which initial HP2 gate opening is followed by binding to Na1 and Na3.

The Na^+^ dependence of HP2 opening rates provides experimental evidence that occupation of the Na1 and Na3 sites modifies the energetics of HP2 opening. Umbrella sampling simulations demonstrated that HP2 assumes mostly an intermediate conformation in *apo* Glt_Ph_, and that Na^+^ binding to only the Na1 site partially promotes the open states, while occupation of both the Na1 and Na3 sites results in almost exclusive adoption of the open state (Fig. 2d). A comparison of a*po*, Na^+^-bound, and Na^+^/l-aspartate-bound Glt_Ph_ crystal structures revealed different orientations of residues around the Na^+^-binding sites, indicating that these residues undergo a series of rearrangements during Na^+^ association. Thus, Na^+^ association starts with the spontaneous opening of HP2, followed by a series of discrete conformational changes that result in maturation of the Na1 and Na3 binding sites: subsequent binding of Na^+^ to Na1 and Na3 stabilizes HP2 in an open state (Fig. 2e).

## HP2 Closure Ensures High Substrate Selectivity in Glutamate Transporters

The flexibility of HP2 allows for exposure of the substrate-binding pocket in the *apo* conformation; thus, aspartate binding might be possible without prior Na^+^ association. However, experimentally obtained EAAT/Glt_X_ transport stoichiometries are constant over a large concentration range [13], demonstrating the existence of mechanisms that prevent substrate association to the Na^+^-free transporter. In unguided MD simulations at physiological [NaCl] and [Asp^−^], aspartate binding also occurred in *apo* and Na1-bound Glt_Ph_; however, Na^+^ occupation of both Na1 and Na3 strongly increased aspartate densities near the aspartate-binding site. These results suggest that electrostatic attraction of aspartate by bound Na^+^ ions effectively stimulates substrate association [49].

HP2 closes after l-aspartate association [45, 61, 64, 65], and tryptophan fluorescence spectroscopy revealed an induced fit mechanism of aspartate binding [64]. Figure 3a shows fluorescence changes upon mixing of L130W Glt_Ph_ with 500 µM l-aspartate and 250 mM Na^+^, as compared to application of l-aspartate to L130W Glt_Ph_ pre-incubated with Na^+^ at the same concentrations. Whereas simultaneous application of l-aspartate and Na^+^ results in slow multi-exponential fluorescence increases, addition of l-aspartate alone to the transporter pre-incubated with Na^+^ caused fluorescence changes proceeding with one rate, that is substantially faster than the largest rate observed upon simultaneous addition of Na^+^ and l-aspartate to the *apo* transporter. The observed rate constants of this process (k_obs_) increase with [l-aspartate] in a saturable fashion (Fig. 3c). This behavior reflects the closure of HP2 after aspartate association, which is the key determinant of substrate selectivity in EAATs/Glts. l-cysteine sulfinic acid binds with a higher affinity than l-aspartate (Fig. 3c) but is transported less effectively (Fig. 3d and e) due to slower closure of HP2 upon l-cysteine sulfinic acid binding compared with l-aspartate binding (Fig. 3c). In contrast, d-aspartate-binding rates saturate at higher values compared with l-aspartate and l-cysteine sulfinic acid (Fig. 3c). Dependence of the HP2 closure rate on the bound substrate establishes that Glt_Ph_ binds amino acids via an induced fit mechanism [64]. This mechanism permits the preferential transport of l-aspartate, which binds with a lower affinity than other substrates and is then released easily on the other membrane side. An induced fit mechanism is thus responsible for high selectivity combined with high effectivity in glutamate transporters.

## Changes in HP2 Dynamics Confer Obligate K^+^ Coupling to EAATs

**Fig. 4:**
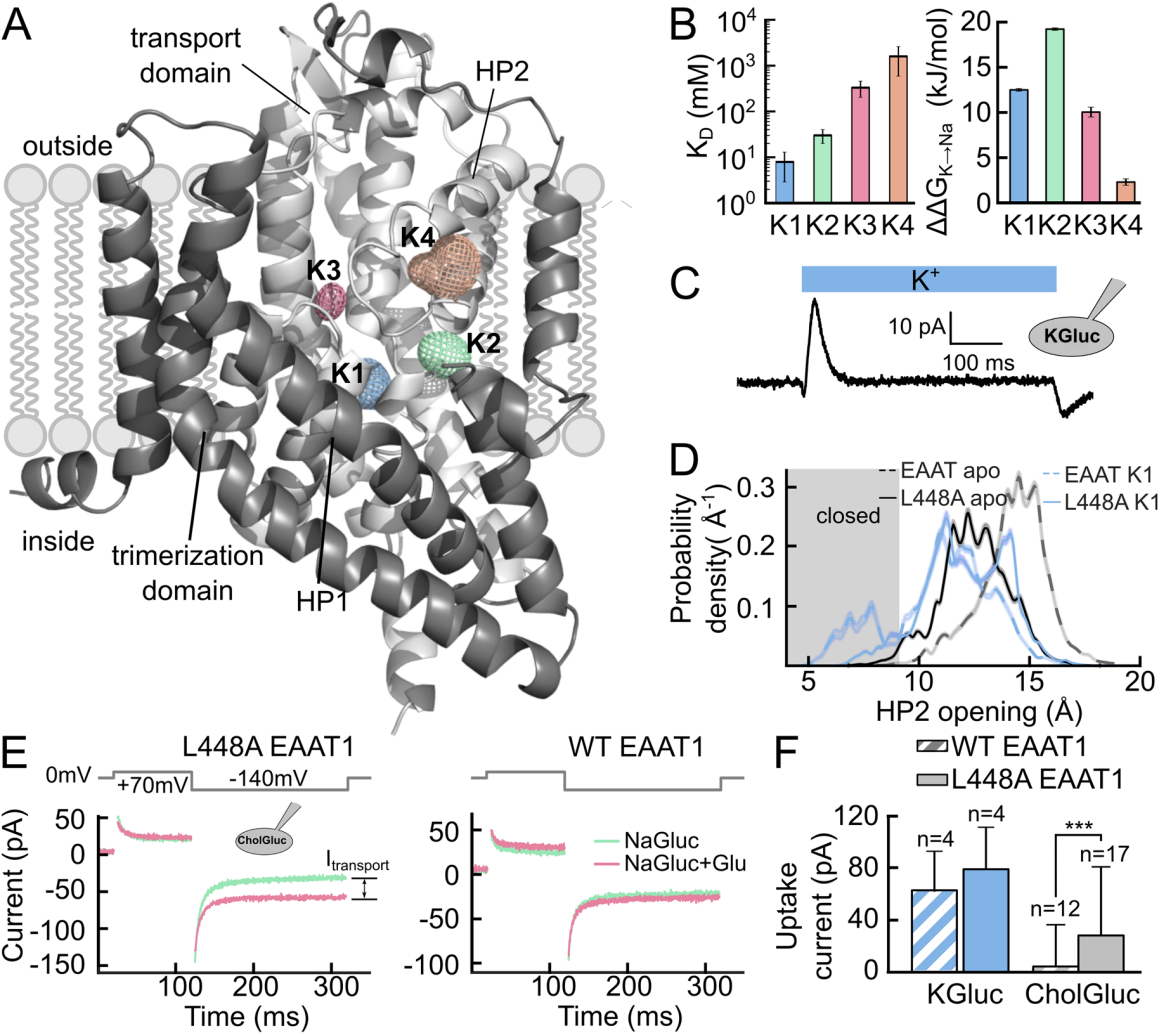
Allosteric gate modulation by K^+^. **a** Isodensity meshes showing the K^+^ density (contoured at 3.5σ) around an EAAT1 monomer in the outward-facing conformation. **b** Left: EAAT1 K^+^ dissociation constants from unguided MD simulations, as obtained by calculating the off rates by second-order rate constants of ion binding. Right: Na^+^/K^+^ selectivities, as calculated from alchemical free-energy simulations. **c** Current responses to a rapid solution exchange from 140 mM CholGluc to 140 mM KGluc for HEK293T cells expressing WT EAAT1 (n = 3). Cells were internally dialyzed with a 115 mM KGluc-based solution and held at 0 mV. **d** Probability density distribution obtained from umbrella sampling simulations for HP2 opening in WT and L448A EAAT1; closed states are indicated by the shaded area. **e** Representative current recordings from cells expressing L448A or WT EAAT1 upon voltage steps to -140 mV before and after superfusion with 1 mM l-glutamate. Cells were internally dialyzed with a K^+^-free solution. **f** Net transport current amplitudes for WT and L448A EAAT1 for different intracellular solutions (***p < 0.001, Student's t‐test). Figure partially reprinted from [68]

K^+^-dependent re-translocation is the final step of the glutamate transport cycle and confers obligatory K^+^ dependence on mammalian EAATs [4, 5]. Since prokaryotic transporters are K^+^ independent [66, 67], an EAAT-specific K^+^-binding site has been assumed—for many years—to have developed late in evolution. Surprisingly, in unbiased MD simulations K^+^ binds to multiple sites in both Glt_Ph_ and EAAT1 [68, 69]. Three of these binding sites (K1, K2, and K3) are identical in Glt_Ph_ and EAAT1, and only K4 is EAAT1-specific (Fig. 4a). However, only K1 and K2 are of sufficiently high affinity and selectivity to serve as binding sites for K^+^-bound translocation: K1 has higher affinity, but lower selectivity than K2 (Fig. 4b) [68]. In simulations, the transport domain with K^+^ bound to K1 carries a negative charge during translocation, whereas occupation of K2 induces the opposite charge movement [68]. In experiments, fast application of K^+^ resulted in the inward translocation of negative charges (Fig. 4c) [70], demonstrating that K1, and not K2, is occupied during translocation in EAATs.

Since K^+^-independent Glt_Ph_ contains the same K^+^-binding sites as obligatory K^+^-coupled EAATs, other mechanisms must be responsible for the distinct transport stoichiometries of mammalian and prokaryotic transporters. In the prokaryotic transporter Glt_Ph_, HP2 can close in the *apo* state [47], so that the transport domain can translocate without bound K^+^ in the *apo* state. In contrast, HP2 is almost always open in *apo* EAAT1, and K^+^ binding is required for EAAT1 HP2 closure and transmembrane translocation. These finding demonstrate that K^+^-independent and obligatory K^+^-coupled transporters differ in their HP2 gate dynamics and predict that mutations in HP2, that render the closed state without bound K^+^ energetically more favorable, can convert K^+^-coupled transporters into K^+^-independent transporters. Figure 4d illustrates HP2 dynamics of one of such mutations, L448A EAAT1, in umbrella sampling simulations. In this mutant, HP2 closure also occurs in the absence of bound K^+^. In experiments, L448A EAAT1 transports glutamate also in the absence of K^+^, demonstrating that changes in HP2 dynamics are sufficient for conferring K^+^ independence to EAAT1 transporters [68] (Fig. 4e).

As the Na^+^- and K^+^-binding sites partially overlap, K^+^ has to be released before Na^+^ can bind. K^+^ cannot dissociate directly from K1 to the bulk solution when HP2 is closed. However, the intrinsic flexibility of HP2 allows for opening with low probability in presence of K^+^ bound to K1, thus permitting K^+^ to dissociate (Fig. 4d). Moreover, K^+^ can move from K1 to K2 and dissociate to the external medium without prior HP2 opening [68]. HP2 opens after K^+^ dissociation, permitting Na^+^ association to Na1 and Na3.

Differences in K^+^ coupling in the EAATs/Glt_X_ are based on distinct allosteric K^+^–HP2 interactions [68, 71], illustrating how allosteric coupling permits modification of transport stoichiometries without the formation of novel binding sites [72, 73]. It may also account for the related ASCTs merely functioning as electroneutral exchangers of neutral amino acids. Small sequence differences in these isoforms may further increase the open probability of HP2 in the *apo* state, such that HP2 closure would only be possible after the binding of Na^+^ and amino acids, thereby converting coupled vectorial transporters into obligatory exchangers [74].

## EAAT Anion Channels Open via Lateral Movement of the Transport Domain

EAATs/Glt_X_ are not only glutamate transporters, but also anion channels that open in response to transitions within the glutamate transport cycle [16, 75–78]. Atomistic MD simulations permitted the identification of an anion-conducting channel conformation [79] that accounts for the experimentally observed lyotropic anion selectivity [14, 80] and unitary current amplitudes in EAATs [81, 82].

**Fig. 5:**
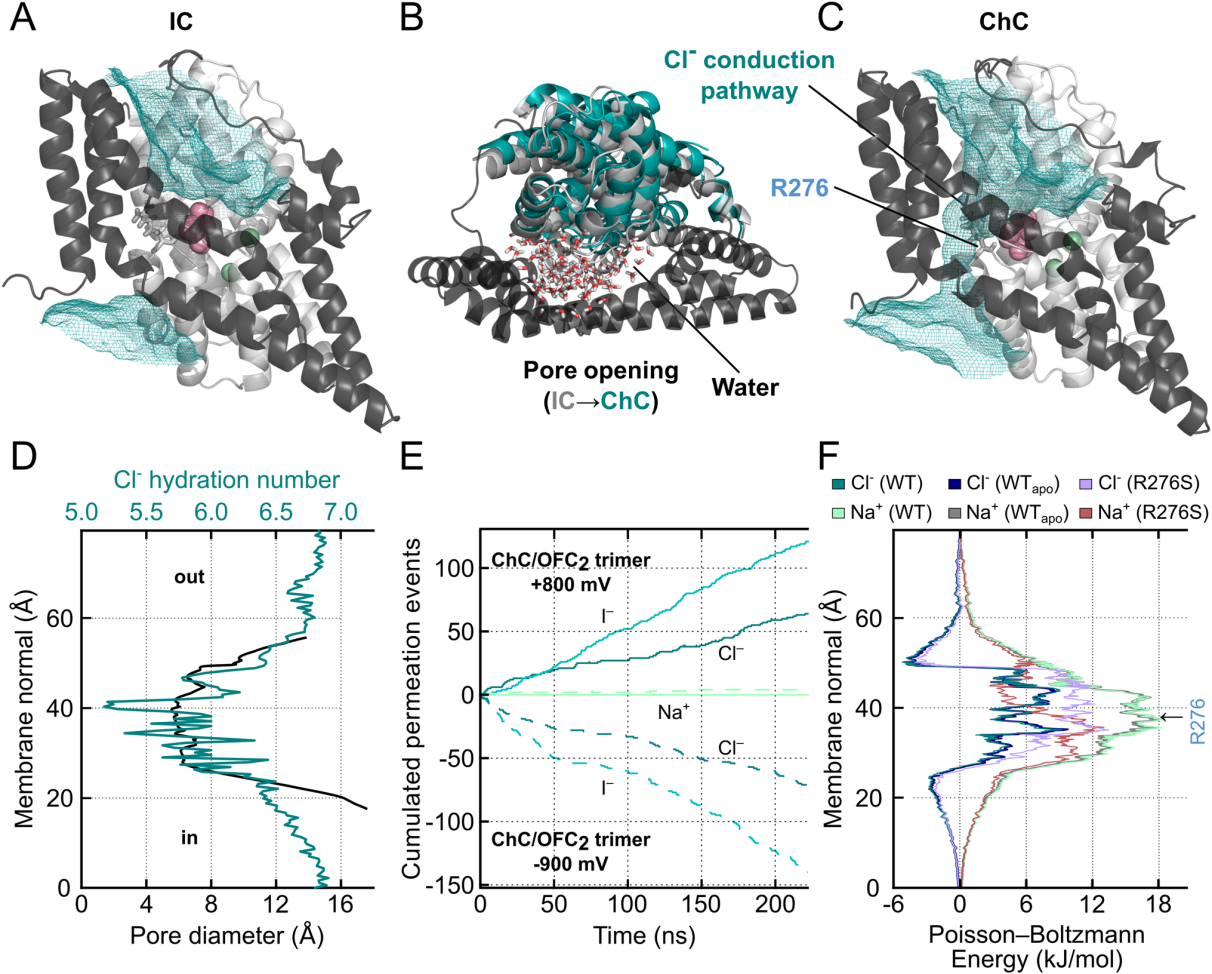
Anion conduction in glutamate transporters. **a** Simulated averaged chloride densities (teal mesh) contoured at 0.2σ at the interface between the transport and trimerization domain in the intermediate conformation (IC; in-between the OFC and IFC) of Glt_Ph_, side view. **b** Conformational changes during anion pore formation, shown as superposition of the IC and open-channel conformation (ChC) onto the static trimerization domains (black) in top view (light gray, transport domain of the IC; teal, transport domain of the ChC) upon application of a membrane potential. Water molecules in the anion conduction pathway in the ChC are shown as sticks. **c** Averaged (teal mesh) chloride distribution in the transport/trimerization domain interface of Glt_Ph_ in the ChC, side view. **d** Pore profiles of the anion pore diameter and Cl^−^ hydration numbers, calculated as the average number of hydrogens within the first Cl^−^ hydration shell. **e** Number of Cl^−^, Na^+^, and I^−^ permeation events through ChC in simulations using either NaCl or NaI solutions at positive or negative transmembrane potentials. **f** Poisson–Boltzmann energies for Na^+^ and Cl^−^ of WT, WT_*apo*_, and R276S Glt_Ph_ in the ChC. Figure partially reprinted from [79]

In all-atom MD simulations of Glt_Ph,_ the OFC and IFC turned out to be non-conductive to ions, with no Cl^−^ permeation even in presence of high transmembrane voltages [79] (Fig. 5a). Furthermore, intermediate conformations—either obtained by X-ray crystallography [51] or by enhanced-sampling simulations of transmembrane translocation [79]—also showed no ion permeation. However, extended unguided simulations starting from such translocation intermediates sampled reversible transitions to a channel-like conformation (ChC) defined by a lateral movement of the mobile substrate transport domain. Transitions to the ChC involved the opening of a cleft between the transport and trimerization domains that permitted frequent Cl^−^ permeation events with partially retained hydration shell after water influx (Fig. 5b and c). Hundreds of simulated anion permeation events defined the conduction pathway with a relatively wide anion pore (minimum diameter of 5.6 Å) and large vestibules on both membrane sites in the ChC (Fig. 5d). Simulations with either NaCl and NaI solutions revealed perfect anion-over-cation selectivity and larger conductance for I^–^ than for Cl^−^ (Fig. 5e). Large anions as glutamate or aspartate were impermeant, and simulated anion permeation rates were consistent with experimental EAAT unitary anion current amplitudes determined by noise analysis. Several amino acids that line the anion pore are hydrophobic, with only one positively charged pore-forming residue, arginine R276 at the tip of HP1. EAATs lack a positive side chain at the position corresponding to R276, but contain an arginine at the position corresponding to M395 in TM8 of Glt_Ph_. This arginine projects its side chain into the same location in the anion conduction pathway and fulfills the same functional role.

The anion conduction pathway in the ChC conformation, as resolved by MD simulations, was tested with fluorescence spectroscopy and cellular electrophysiology experiments [79]. Tryptophan fluorescence is collisionally quenched by I^−^, and tryptophans inserted close to the simulated conduction pathway showed a significant decrease in fluorescence upon the application of I^−^, whereas regions remote from the simulated anion pore were not accessible to I^−^. As predicted from MD simulations, R276 dominates the anion selectivity of the conduction pathway and neutralization of this residue caused a loss of anion-over-cation selectivity in simulations and experiments (Fig. 5f). An in silico mutagenesis screen of all pore-forming mutants identified residues that change unitary current amplitudes and/or permit cation permeation. All substitutions that modified the anion conduction pathway in simulations affected unitary current amplitudes or Na^+^-to-Cl^−^ permeation in experiments. These data demonstrate that EAATs/Glt_Ph_ can adopt the anion-conducting conformation identified by MD simulations under experimental conditions. The computationally predicted channel conformation and conduction mechanism account for all published mutagenesis results on EAAT anion channels [83–85].

Electrophysiological experiments demonstrated that EAAT anion channel opening upon rapid substrate application is delayed compared with glutamate translocation [86]. This suggests that anion-conducting states are located outside the main transport cycle and can only be reached via branching transitions from the translocation pathway. However, crystallographic studies and single-molecule fluorescence resonance energy transfer (FRET) experiments on Glt_Ph_ [87] suggested that the identified lateral movement of the transport domain might always occur during elevator translocation and, therefore, that the ChC might be visited in every transport cycle. Thus, although the molecular details of anion conduction in glutamate transporters are well understood, further functional and computational research is needed to understand the exact relationship between substrate translocation and channel opening.

## HP2 is a Master Key of Glutamate Transporter Function

EAATs are amazingly complex transport proteins. They couple glutamate uptake to the symport of 3 Na^+^ and 1 H^+^, in counter transport with 1 K^+^, over a large range of concentrations [13, 21, 22]. They are trimers [88, 89], and each of the subunits can function independently from its neighbors [90–93]. Lastly, EAATs are not only secondary-active transporters, but also anion channels.

**Fig. 6:**
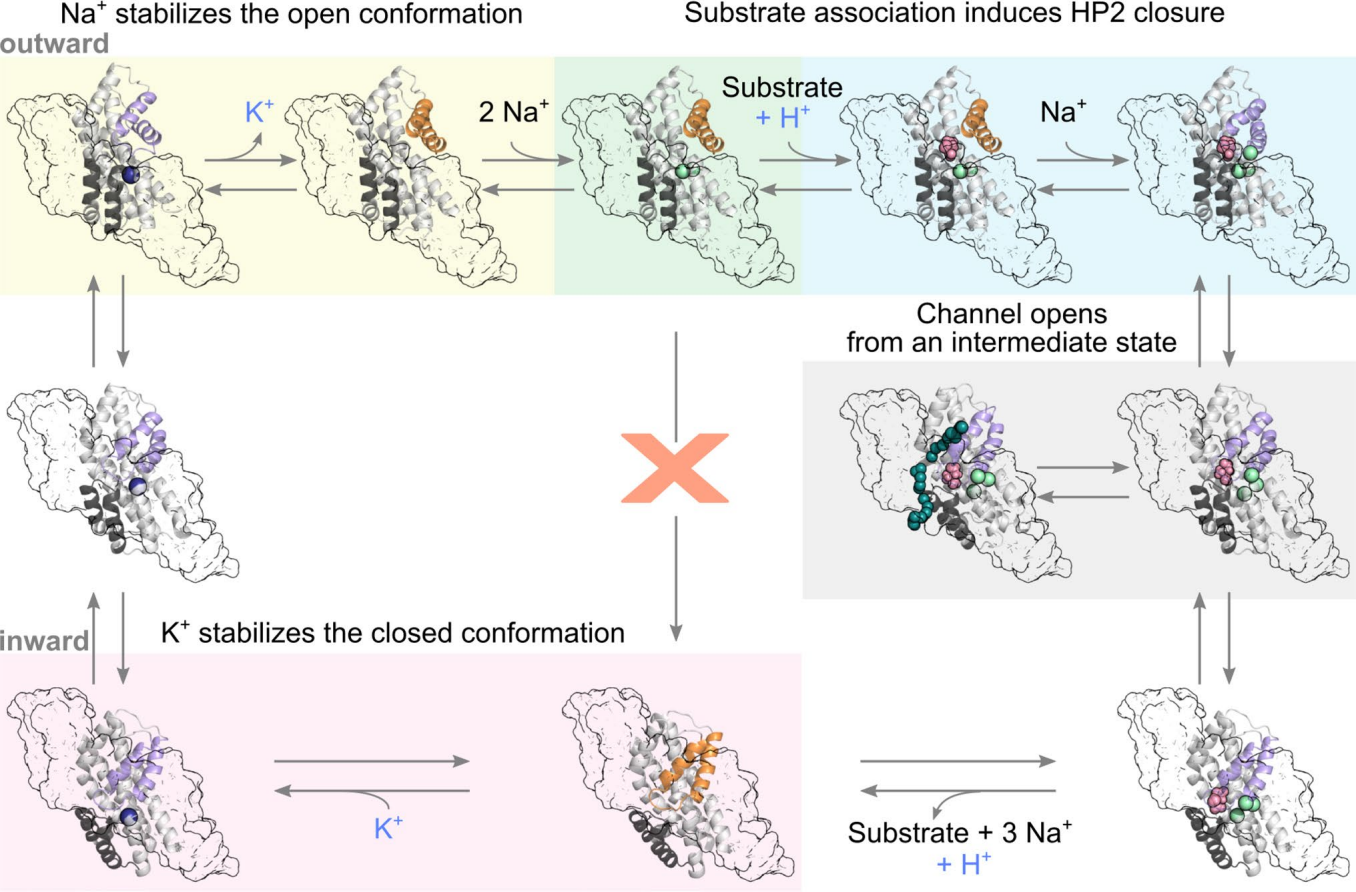
Role of HP2 in the various EAAT transport functions. Depiction of the EAAT/Glt_X_ transport cycle. From upper left: the cycle starts in the *apo* state for Glt_X_ and in the K^+^-bound state for EAATs, HP2 is closed (PDB ID: 4OYE, K^+^ position from [68]). In the next step, K^+^ is released (in EAATs) and HP2 opens, followed by the binding of two Na^+^ ions (PDB ID: 7AHK). Association of the substrate follows, together with the binding of one H^+^ in EAATs, induces HP2 closure, which permits elevator translocation (PDB ID: 2NWX, Na3 position from PDB ID: 7AHK). Translocation intermediates can open an anion-selective pore to allow thermodynamically uncoupled anion currents. From the inward closed state (PDB ID: 3KBC, Na3 position from PDB ID: 7AHK), the ligands are released upon HP2 opening. Finally, HP2 closes once again in the *apo* state for Glt_X_ and in the K^+^-bound state in EAATs to enable re-translocation (PDB ID: 3KBC, K^+^ position from [68])

For almost all of these functions, HP2 has emerged as a key structural element (Fig. 6). Translocation can only occur after closure of HP2; thus, stabilization of the open or closed state by distinct combinations of bound ions and substrates ensures transport at a fixed stoichiometry. In Glt_Ph_, the internal flexibility of HP2 enables re-translocation in the *apo* state [67, 68] and transitions between the OFC and IFC in the absence of ligands [59]. HP2 must close to permit translocation and open to allow the effective release of substrates/ions and the association of new substrates/ions [58]. Due to its inherently flexible nature, HP2 switches between the open and closed states. Simultaneous occupation of Na1 and Na3 keeps HP2 open to permit amino acid association, which is followed by Na2 occupation and HP2 closure. Moreover, HP2 is crucial for substrate selectivity in this class of transporters. The main substrates of EAATs/Glt_X_—l-glutamate/l-aspartate—have lower binding affinities compared with other substrates and, thus, effectively dissociate on the other membrane site. They are translocated effectively because they promote HP2 closure. This induced fit mechanism permits a combination of high selectivity and effective l-glutamate/l-aspartate transport by EAATs/Glt_X_.

We have wondered for decades how EAAT glutamate transporters mediate multiple transport functions, with their strict transport stoichiometry and tightly regulated anion channel gating and selectivity. Recent evidence demonstrated a surprising functional simplicity, with only few key processes responsible for this complexity. Substrates are moved across the membrane by shuttling the transport domain from the OFC to the IFC. The transport domain encompasses distinct binding sites that partially overlap and allosterically regulate HP2 opening and closing transitions, which in turn regulate access to some of these binding sites. This permits a total of six ions to be moved within a single transport cycle at fixed stoichiometry, with a clearly defined binding order, high selectivity, and highly efficient loading and unloading. In addition, this process permits the opening and closing of a selective anion channel whose physiological and pathophysiological importance we are just starting to appreciate.

## Abbreviations

ChC: Channel-like conformation
EA6: Episodic ataxia 6
EAAT: Excitatory amino acid transporter
Glt_Ph_: Glutamate transporter homologue from *Pyrococcus horikoshii*
Glt_Tk_: Glutamate transporter homologue from *Thermococcus kodakarensis*
IC: Intermediate conformation
IFC: Inward-facing conformation
MD: Molecular dynamics
OFC: Outward-facing conformation
